# A Free-Standing α-MoO_3_/MXene Composite Anode for High-Performance Lithium Storage

**DOI:** 10.3390/nano12091422

**Published:** 2022-04-21

**Authors:** Zihan Guo, Dong Wang, Zhiwei Wang, Yanfang Gao, Jinrong Liu

**Affiliations:** School of Chemical Engineering, Inner Mongolia University of Technology, Hohhot 010051, China; gzh@imut.edu.cn (Z.G.); wangd0419@163.com (D.W.); zhiwei2009@126.com (Z.W.)

**Keywords:** free-standing electrode, α-MoO_3_/MXene composite anode, high capacity, electrochemical energy storage

## Abstract

Replacing the commercial graphite anode in Li-ion batteries with pseudocapacitor materials is an effective way to obtain high-performance energy storage devices. α-MoO_3_ is an attractive pseudocapacitor electrode material due to its theoretical capacity of 1117 mAh g^−1^. Nevertheless, its low conductivity greatly limits its electrochemical performance. MXene is often used as a 2D conductive substrate and flexible framework for the development of a non-binder electrode because of its unparalleled electronic conductivity and excellent mechanical flexibility. Herein, a free-standing α-MoO_3_/MXene composite anode with a high specific capacity and an outstanding rate capability was prepared using a green and simple method. The resultant α-MoO_3_/MXene composite electrode combines the advantages of each of the two components and possesses improved Li^+^ diffusion kinetics. In particular, this α-MoO_3_/MXene free-standing electrode exhibited a high Li^+^ storage capacity (1008 mAh g^−1^ at 0.1 A g^−1^) and an outstanding rate capability (172 mAh g^−1^ at 10 A g^−1^), as well as a much extended cycling stability (500 cycles at 0.5 A g^−1^). Furthermore, a full cell was fabricated using commercial LiFePO_4_ as the cathode, which displayed a high Li^+^ storage capacity of 160 mAh g^−1^ with an outstanding rate performance (48 mAh g^−1^ at 1 A g^−1^). We believe that our research reveals new possibilities for the development of an advanced free-standing electrode from pseudocapacitive materials for high-performance Li-ion storage.

## 1. Introduction

With the flourishing of electric vehicles and portable electronic devices, there is a growing demand for high-performance lithium-ion batteries. Anode materials play a key role in such batteries, yet designing new anode materials that can overcome the limitations of the graphite anodes most frequently used today remains a significant challenge. These limitations include a low theoretical capacity (372 mAh g^−1^) and an insufficient rate capability [1,2]. Recently, some transition-metal oxides (such as T-Nb_2_O_5_ [3,4], MoO_3_ [5,6], and V_2_O_5_ [7,8]) and two-dimensional (2D) metal carbide materials (MXene) [9,10] were found to exhibit ion intercalation pseudocapacitance behavior in a nonaqueous Li-ion electrolyte solution. In contrast to the slow and diffusion-controlled intercalation of battery-type materials, pseudocapacitive materials store energy via a rapid and surface-controlled Faraday reaction [11,12,13]. In addition, researchers hope to develop a new electrode without conductive agents, adhesives, or collectors in a simple and green way, as the goal of carbon neutrality has gained worldwide attention [14]. Therefore, replacing the commercial graphite anode of Li-ion batteries with pseudocapacitor materials and basing a unique structure on it is one way to obtain high-performance energy storage devices.

As an pseudocapacitive material, MoO_3_ is widely sought-after because of its rich resources, low cost, and ultrahigh theoretical capacity of 1117 mAh g^−1^ [15,16]. In particular, α-MoO_3_ has a unique and stable 2D layered structure, which allows for the rapid intercalation and deintercalation of ions between its layers [17,18]. Nevertheless, MoO_3_ has an inherently low conductivity and modest reaction kinetics, which lead to an inferior rate capability and a rapid capacity decay during charge storage [15,17]. An efficient strategy to overcome these obstacles is to construct an interconnected conductive network among numerous MoO_3_ nanounits. MXene (Ti_3_C_2_) is especially suitable as the 2D conductive substrate of MoO_3_ because of its unparalleled electronic conductivity and lithium transport ability [10,19,20]. Furthermore, MXene has excellent mechanical flexibility, so it could be used as a flexible framework to develop a MoO_3_ non-binder electrode [21,22]. Nevertheless, the re-stacking of MXene is an important technological problem for the electrode structure. It has been proven that the incorporation of metal oxide nanomaterials into the MXene interlayer can produce a “spacer effect”, preventing effectively re-stacking and increasing the layer space and the number of surface active sites [2,18]. In previous research, Zheng et al. [18,23] and Wang et al. [24] constructed an independent MoO_3_/Ti_3_C_2_ electrode using a vacuum filtration method, and it showed an excellent rate capability as a supercapacitor electrode material in aqueous electrolytes. However, the small voltage window of the aqueous electrolytes limited the maximum energy storage capability of the MoO_3_/Ti_3_C_2_ electrode. Zhang et al. [17] also fabricated a MoO_3_/Ti_3_C_2_ heterostructure through simple vacuum filtration and prepared a traditional slurry-casted electrode that showed an excellent lithium-ion storage performance in organic electrolyte. Nevertheless, the traditional slurry-casted electrode requires binders and conductive agents, which do not contribute to capacity.

The results of the research above convinced us that constructing α-MoO_3_/MXene composite films to be directly used as free-standing electrodes would be an efficient strategy to improve the lithium-ion storage performance of α-MoO_3_. We thus fabricated α-MoO_3_/MXene composite films by mixing prefabricated α-MoO_3_ nanobelts and MXene nanosheets in an aqueous solution under ultrasonication, followed by using a simple vacuum filtration method. As expected, the resultant α-MoO_3_/MXene composite electrode was able to combine the advantages of each of the two components; in particular, it possessed an enhanced electronic conductivity and improved Li^+^ diffusion kinetics owing to the formation of a three-dimensional network structure, thus greatly improving its lithium-ion storage performance. This free-standing electrode delivered a high discharge specific capacity (1008 mAh g^−1^ at 0.1 A g^−1^), an outstanding rate capability (172 mAh g^−1^ at 10 A g^−1^), and an excellent cycle stability (500 cycles at 0.5 A g^−1^). Based on a commercial LFP cathode and an α-MoO_3_/MXene anode, the constructed Li-ion full cell exhibited a high discharge specific capacity of 160 mAh g^−1^ at 0.02 A g^−1^, a superior rate capability of 48 mAh g^−1^ at 1 A g^−1^, and a remarkable cycling stability. The α-MoO_3_ non-binder electrode with MXene as its conductive substrate and flexible framework, it was used for the first time as the anode of a lithium-ion battery. This research fills the research gap for an α-MoO_3_/MXene non-binder electrode in a Li-ion organic electrolyte and opens up new possibilities for the development of advanced free-standing electrodes from other pseudocapacitive materials for Li-ion storage.

## 2. Materials and Methods

### 2.1. Preparation of α-MoO_3_ Nanobelts

α-MoO_3_ nanobelts were prepared by controlling the molar ratio of H^+^ to Na^+^ (1.5:1) to ensure the formation of the α-MoO_3_ phase [25]. The typical process was as follows: A total of 5 mmol of Na_2_MoO_4_·2H_2_O (Aladdin, China) and 10 mmol of NaCl (Aladdin, China) were dissolved in 40 mL deionized water, and 10 mL of 3 M HCl was added dropwise under magnetic stirring. After stirring for 2 h, the solution obtained was transferred to a 100 mL Teflon-lined autoclave. The autoclave was heated to 180 °C for 24 h and then left to cool down to room temperature. The as-prepared sample was collected by centrifugation; washed with deionized water and ethanol, respectively, 3 times; and dried at 80 °C overnight.

### 2.2. Preparation of MXene Nanosheets

Ti_3_C_2_ MXene was synthesized through etching the Al component of Ti_3_AlC_2_ (Xi’an Qiyue Biotechnology Co., Ltd., Xi’an, China) MAX phase using fluoride-based salts [11,26]. The etchant was prepared by adding 1 g of LiF (Aladdin, China) to 20 mL 9 M of HCl. Then, 1 g of Ti_3_AlC_2_ was slowly added to the above etchant with stirring in an ice bath. The stirring was kept at 35 °C for another 24 h. The solid product was washed and centrifuged several times with deionized water (5 min per cycle at 3500 rpm) until pH ≈ 6. The precipitate was collected and dispersed in the deionized water. After ultrasonic treatment in an ice bath under flowing argon for 1h, it was centrifuged at 3500 rpm for 1h. The supernatant was collected to obtain the few-layered MXene suspension. Finally, the few-layered MXene nanosheets were obtained by freeze drying for three days.

### 2.3. Preparation of α-MoO_3_/MXene Film Free-Standing Electrode

The α-MoO_3_/MXene film was prepared by optimizing the previous method [11,27], as shown in Figure S1. A total of 1.0 mg mL^−1^ of α-MoO_3_ aqueous solution and 1.0 mg mL^−1^ of MXene aqueous solution were mixed according to a mass ratio of 3:1 and ultrasonically treated for 10 min under flowing argon. The MoO_3_/MXene films were obtained through the vacuum filtration of uniform and stable suspensions of MoO_3_ and Ti_3_C_2_, and they were then dried in vacuum at 40 °C for 12 h. The free-standing electrode with a diameter of 1.1 mm was subsequently obtained and dried in vacuum at 105 °C overnight before assembling CR2032 coin-type cells.

### 2.4. Material Characterization

The morphology of the samples was characterized with a scanning electron microscope (SEM, SU8220, Hitachi, Naka, Japan) and a transmission electron microscope (TEM, Talos F200X, FEI, Brno, Czech). X-ray photoelectron spectroscopy (XPS) was performed with a ESCALAB 250 Xi photoelectron spectrometer (Thermo-Fisher, America) equipped with a monochromatic Al X-ray source (1486.6 eV), and the XPS binding energy values were calibrated by fixing the C-1s core level data of additional carbon at 284.8 eV. X-ray diffraction (XRD) was carried out on a Empyrean diffractometer (PANalytical B. V., Holland) equipped with a Cu K-alpha radiation source (λ = 1.5406 Å). Raman spectroscopy was performed using a laser excitation of 532 nm (InVia Microscope Raman, Renishaw, England).

### 2.5. Electrochemical Measurements

For the preparation of the pure MoO_3_ anode, MoO_3_ (80 wt.%), acetylene black (10 wt.%), and polyvinylidene fluoride (PVDF, 10 wt.%) were mixed in nitromethyl pyrrolidone (NMP) to produce a homogeneous slurry. The mixture was then uniformly coated onto a Cu foil and finally dried in a vacuum oven at 105 °C overnight. The LiFePO_4_ cathode was prepared using the same method, except that the slurry was coated on Al foil instead. All the electrochemical tests were carried out on CR2032 coin cells in an argon-filled glove box (H_2_O and O_2_ < 0.1 ppm), and the half battery was assembled with lithium metal foil as the counter and reference electrode. Porous polypropylene membrane (Celgard 2500, America) was used as a diaphragm, and 1.0 M of LiPF_6_ dissolved in ethylene carbonate/diethyl carbonate (EC/DEC, 1:1 *v*/*v*) was employed as the electrolyte.

Cyclic voltammetry (CV) measurements were performed on a CHI760E electrochemical station (Shanghai, China). The electrochemical impedance spectroscopy (EIS) measurements were obtained using a PARSTAT3000A-DX electrochemical station (Princeton, NJ, USA) with an AC amplitude of 10 mV in the frequency range of 0.01 Hz–100 kHz. Galvanostatic charge/discharge (GCD) tests were carried out using a Land CT2001A and a G340A model battery test system (Wuhan, China).

## 3. Results and Discussion

### 3.1. Microstructure Characterization

**Figure 1:**
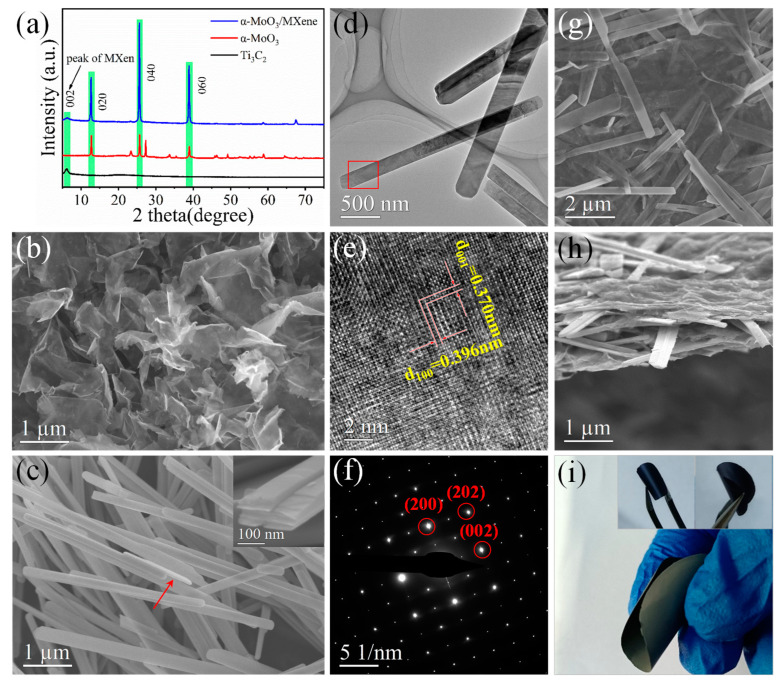
(**a**) XRD diffraction patterns of Ti_3_C_2_, MoO_3_, and MoO_3_/MXene. (**b**) SEM image of Ti_3_C_2_. (**c**) SEM image of α-MoO_3_. (**d**) TEM image of α-MoO_3_. (**e**) HR-TEM image of α-MoO_3_. (**f**) SAED pattern of α-MoO_3_. (**g**,**h**) SEM images of α-MoO_3_/MXene. (**i**) Photographs of free-standing α-MoO_3_/MXene film and electrode.

The overall preparation procedure of the α-MoO_3_/MXene film is schematically illustrated in Figure S1. Flexible and free-standing α-MoO_3_/MXene films were obtained by vacuum filtrating the homogenous mixed dispersion. It is worth noting that the preparation of the free-standing films was carried out at room temperature, effectively avoiding the oxidation of the MXene nanosheets under high-temperature conditions. It can be seen from the X-ray diffraction (XRD) pattern (Figure S2) that the characteristic peak (104) of Ti_3_AlC_2_ located at *2θ* = 39° completely disappeared, indicating the effective removal of the Al component, and an obvious shift in the (002) peak from 9.5° to 6.2° can also be seen, corresponding to a d-spacing of 14.24 Å. These results are consistent with those from previous reports, indicating that the MXene nanosheets were successfully exfoliated [22,28]. SEM showed that the MXene nanosheets had a clean surface, as shown in Figure 1b. The 2D lamellar structure of flakes was further observed through transmission electron microscopy (TEM) (Figure S3a) to consist of well-defined edges and a lateral dimension of hundreds of nanometers, which indicates that the prepared MXene nanosheets were of a high quality. There were two obvious diffraction rings of (110) and (006) facets in the overall selected area electron diffraction (SAED) pattern, as shown in an inset of Figure S3a, revealing the polycrystalline nature of Ti_3_C_2_ [29,30].

Similarly, the XRD pattern of the as-prepared MoO_3_ nanobelts in Figure 1a (red) exhibited sharp peaks, which were attributed to the orthorhombic phase (α-MoO_3_ phase, JCPDS No. 05-0508) [25,31]. The SEM image and low-magnification TEM image in Figure 1c and Figure 1d, respectively, confirmed that most of the α-MoO_3_ exhibited uniform belt-like nanostructures, and the nanobelts were 2D layered structures with lengths of 4~8 µm and widths of 200~300 nm. It can be seen in Figure 1c that the α-MoO_3_ nanobelts were found to be about 60 nm in thickness. The high-resolution TEM (HR-TEM) image (Figure 1e) was acquired at a rectangular position, as shown in Figure 1d. The image showed two sets of crystal lattice fringes with d-spacings of 0.396 and 0.370 nm, corresponding to the (100) and (001) planes of the α-MoO_3_ phase (JCPDS No. 05–0508), respectively, which is in accordance with the XRD result. Moreover, the spot pattern in the selected area electron diffraction (SAED) image indicated the single-crystalline nature of α-MoO_3_ (Figure 1f) [15].

The XRD pattern of the α-MoO_3_/MXene films (Figure 1a, blue) only exhibited the typical peaks for α-MoO_3_ and Ti_3_C_2_, meaning that no new phases were created when the two components were mixed. The SEM images of the α-MoO_3_/MXene films indicated the randomly oriented and interconnected nanostructure, with α-MoO_3_ nanobelts dispersed between the MXene nanosheets (Figure 1g,h). This significantly alleviated the re-stacking of the Ti_3_C_2_ nanosheets, thereby enlarging the accessible active surface for energy storage. This, in turn, effectively established a well-conducting network for α-MoO_3_ nanoribbons, thus significantly enhancing the extrinsic electrical conductivity of α-MoO_3_ and facilitating the rapid transport of electrons. The HAADF and TEM images of the α-MoO_3_/MXene composite revealed that the α-MoO_3_ nanobelts were dispersed on the surface of the Ti_3_C_2_ nanosheets, and the conductive Ti_3_C_2_ nanosheets served as a bridge for the α-MoO_3_ nanobelts, thus allowing for rapid and efficient electron transfer between the α-MoO_3_ nanobelts and the Ti_3_C_2_ nanosheets during the rapid charge/discharge process (Figure S3b,c). The energy dispersive X-ray spectroscopy (EDS) element mapping images of Ti, C, O, and Mo confirmed that these four elements were homogeneously present in the α-MoO_3_/MXene film (Figure S3d). At the same time, obvious polycrystalline diffraction rings of Ti_3_C_2_ and a single-crystalline dot pattern for α-MoO_3_ were present in the SAED patterns of the α-MoO_3_/MXene film (inset of Figure S3b). This showed that the structure of the two components did not change after mixing, which is consistent with the XRD results. Digital photos of the α-MoO_3_/MXene free-standing electrode are displayed in Figure 1i. Despite not using any binders, all the films exhibited an excellent flexibility and a high degree of deformation, such that they could be curled many times without cracking.

**Figure 2:**
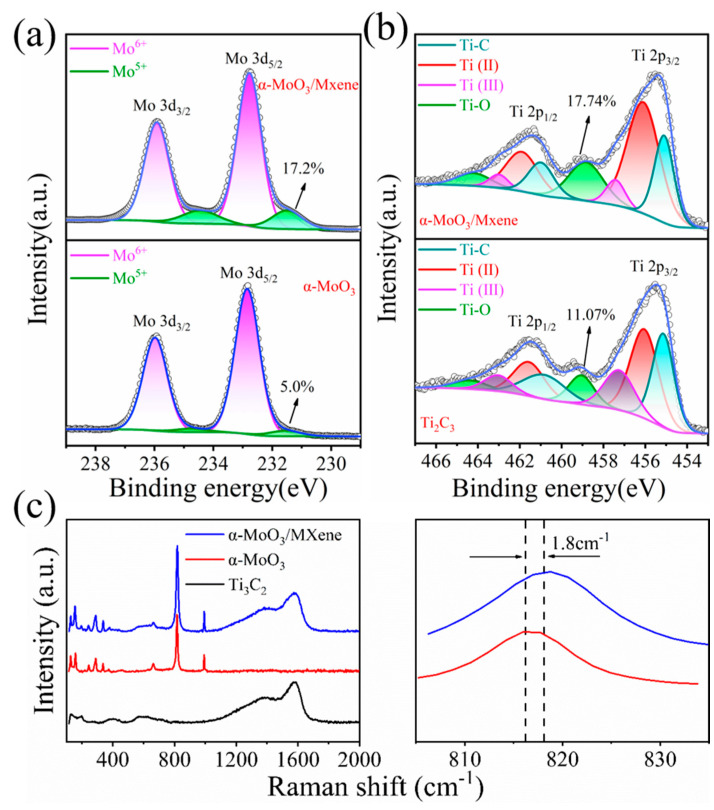
(**a**) Mo 3d XPS spectra of α-MoO_3_ and α-MoO_3_/MXene. (**b**) Ti 2p XPS spectra of Ti_3_C_2_ and α-MoO_3_/MXene. (**c**) Raman spectra of Ti_3_C_2_, α-MoO_3_, and α-MoO_3_/MXene.

X-ray photoelectron spectroscopy (XPS) was applied to further characterize the surface electronic states of the α-MoO_3_/MXene film. The Mo 3d core-level XPS spectra of the pure α-MoO_3_ and the α-MoO_3_/MXene composite showed that the two peaks could be well fitted with an intensive Mo^6+^ doublet and a weak Mo^5+^ doublet (Figure 2a). The doublets centered at 232.9 and 236.0 eV could be assigned to the Mo 3d_5/2_ and Mo 3d_3/2_ orbital electrons of Mo^6+^ for α-MoO_3_ [15], respectively, and the peaks at 231.6 and 234.6 eV corresponded to the 3d_5/2_ and 3d_3/2_ orbital electrons of Mo^5+^ [32], respectively. Notably, the amount of Mo^5+^ in the α-MoO_3_ increased from 4.76% for pure α-MoO_3_ to 14.64% for the composite. The Ti 2p core-level spectrum could be divided into four doublets with Ti 2p_3/2_ centered at 455.1, 456.1, 457.4, and 458.8 eV, as shown in Figure 2b; these could be assigned to the Ti-C, Ti(II), Ti(III), and Ti-O bonds [17,22], respectively. Similarly, after mixing the suspension of α-MoO_3_ and Ti_3_C_2_, it was obvious that the fraction of Ti^4+^ in Ti_3_C_2_ increased from 11.07% for pure Ti_3_C_2_ to 17.74% for the composite. Based on these results, when the two suspensions were mixed, the fraction of Mo^6+^ in α-MoO_3_ was reduced by Ti_3_C_2_, which is able localize to form Mo^5+^ centers and oxygen vacancies [18,20,23]. This somewhat unexpected result is beneficial to enhance the intrinsic conductivity of α-MoO_3_, because it has been confirmed that partially reduced α-MoO_3_ with oxygen vacancies has a better electrochemical performance compared with pure α-MoO_3_ [4,15,17,32,33]. The Raman spectra of the Ti_3_C_2_, α-MoO_3_, and α-MoO_3_/MXene films are shown in Figure 2c. The characteristic peaks for Ti_3_C_2_ and α-MoO_3_ were maintained after the two suspensions were mixed. The slight blue shift in the Raman band at 816.7 cm^−1^ for the α-MoO_3_/MXene film compared with that of the pristine α-MoO_3_ also confirmed the presence of oxygen vacancies [17,29]. This result is almost completely consistent with that of the above XPS analysis. These oxygen vacancies were considered to help improve the conductivity of the composites, giving rise to the rapid transport of electrons during the charge/discharge processes.

### 3.2. Electrochemical Characterization

**Figure 3:**
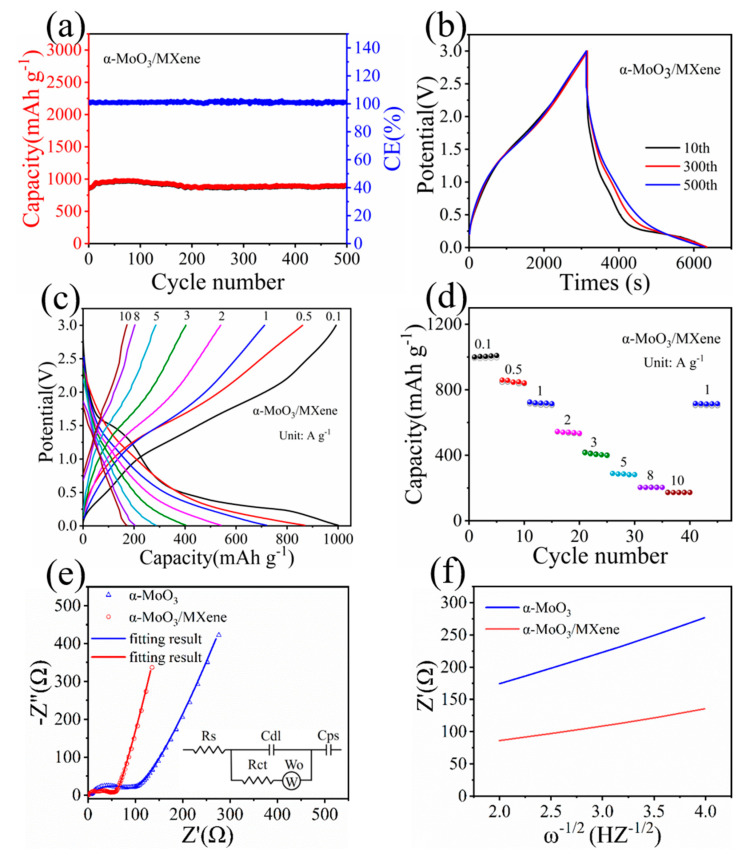
(**a**) Cycling performance of α-MoO_3_/MXene at 0.5 A g^−1^. (**b**) GCD curves of α-MoO_3_/MXene in different cycles at 0.5 A g^−1^. (**c**) GCD curves and (**d**) rate capability of α-MoO_3_/MXene. (**e**) Nyquist plots of α-MoO_3_ and α-MoO_3_/MXene (inset of electrical equivalent circuit). (**f**) The relationship between Z′ and ω^−1/2^ (ω = 2πf) in the low-frequency region of α-MoO_3_ and α-MoO_3_/MXene.

The as-prepared α-MoO_3_/MXene films were flexible and directly used as electrodes without the addition of any conductive agent, adhesive, or collector. The long-term cyclic stability of the α-MoO_3_/MXene free-standing electrode was first evaluated at a relatively high current density of 0.5 A g^−1^, as shown in Figure 3a. The α-MoO_3_/MXene free-standing electrode exhibited an initial reversible capability of 800 mAh g^−1^. It is noteworthy that this electrode could exhibit an ultrahigh discharge capacity of 903 mAh g^−1^ after 500 cycles, much higher than the α-MoO_3_ electrode (Figure S4a). The α-MoO_3_ electrode exhibited a rapid rise in discharge specific capacity from 355 to 542 mAh g^−1^ in the first 15 cycles, followed by a rapid decline in discharge specific capacity to an exceedingly low value of 106 mAh g^−1^ after 500 cycles. Figure 3b and Figure S4b show the galvanostatic charge/discharge (GCD) curves of the α-MoO_3_/MXene and α-MoO_3_ electrodes, respectively, at 0.5 A g^−1^, which is consistent with the above long-term cycle analysis. Compared to the GCD curves of the two electrodes in the 10th cycle, the GCD curve of the α-MoO_3_ electrode was found to have obviously deviated from its ideal triangular shape, and the GCD curve of the α-MoO_3_/MXene electrode exhibited a quasi-triangular shape without an obvious IR drop. With the increase in the number of cycles, the capacity of the α-MoO_3_ electrode declined rapidly, and the shape of the curve also changed significantly. However, compared to the GCD curves of the α-MoO_3_/MXene electrode in the 10th, 300th, and 500th cycle, the shape changed little. The above results indicate that the α-MoO_3_/MXene electrode had a high capacity and an excellent cycle stability. This can be attributed to the rapid transfer of electrons and the rapid intercalation/delamination of electrolyte ions. In addition, the rate performance was further tested at different current densities. Figure 3c,d exhibit the specific capacity of the α-MoO_3_/MXene composite at various current densities. The α-MoO_3_/MXene electrode exhibited high discharge capacities of 1008, 857, 718, 540, 409, 285, 203, and 172 mAh g^−1^ at current densities of 0.1, 0.5, 1, 2, 3, 5, 8, and 10 A g^−1^, respectively. Remarkably, it was still able to exhibit a high specific capacity of 172 mAh g^−1^ when the current density was returned to 10 A g^−1^, indicating its good rate capability. This result is encouraging, considering that our α-MoO_3_/MXene electrode is entirely binder-free and carbon-free. Importantly, the specific capacity and rate performances of the MoO_3_/MXene electrode were highly similar or even superior to those of the previously reported Mo-based electrodes for Li-ion batteries (Figure S5). This demonstrates that the introduction of MXene nanosheets could allow for the construction of a 3D conducting network for individual α-MoO_3_ nanobelts, which could significantly improve the electronic conductivity. At the same time, the α-MoO_3_ nanobelts acted as “spacers” between the MXene nanosheets to prevent re-stacking, increase the electrochemical active surface area, and shorten the diffusion/transport paths of electrons and ions.

Electrochemical impedance spectroscopy (EIS) was further performed to analyze the reaction kinetics of the α-MoO_3_/MXene electrode. As can be seen in Figure 3e, the Nyquist plots consist of a quasi-semicircle in the high-frequency area and a straight line in the low-frequency area. The equivalent circuit (inset of Figure 3e) consisted of the solution resistance (R_s_), charge-transfer resistance (R_ct_), interfacial diffusive resistance (Warburg impedance, W_0_), double-layer capacitance (C_dl_), and a faradic pseudocapacitor (C_ps_) [34]. In the high-frequency area, the diameter of the semicircle presented the charge transfer resistance R_ct_. According to the equal circuit fitting, the R_ct_ value of the α-MoO_3_/MXene electrode (45.1 Ω) was much smaller than that of the α-MoO_3_ electrode (87.5 Ω) as a result of the introduction of MXene nanosheets, which obviously enhanced the electrical conductivity and facilitated the rapid charge transfer. In addition, the much larger linear slope of the α-MoO_3_/MXene electrode in the low-frequency area compared with the α-MoO_3_ electrode implied a rapid solid-state diffusion of Li^+^ ions for the α-MoO_3_/MXene electrode [15,35]. Additionally, as can be seen from the relationship between Z′ and ω (ω = 2πf) in the low-frequency area (Figure 3f), the α-MoO_3_/MXene electrode exhibited a much lower slope than the α-MoO_3_ electrode, resulting in better ion diffusion/transport kinetics [22]. This could be attributed to the structure of the α-MoO_3_/MXene electrode, which may have promoted the penetration and diffusion/transport of electrolyte ions [4]. These results are in good agreement with those indicated in the Nyquist plots.

**Figure 4:**
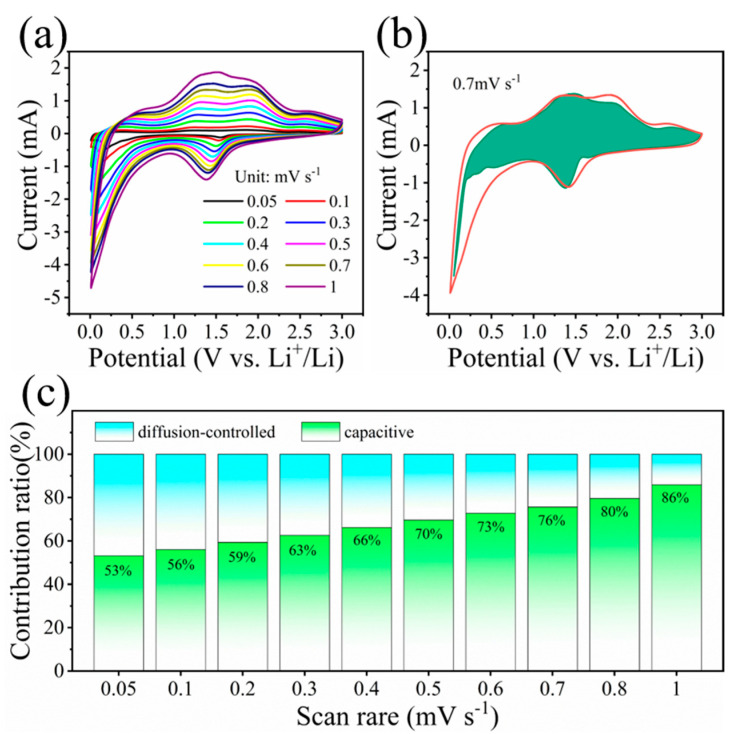
(**a**) CV curves of α-MoO_3_/MXene ranging from 0.05 mV s^−1^ to 1 mV s^−1^. (**b**) Capacitive charge storage contribution (green) at 0.7 mV s^−1^. (**c**) Capacitive contribution ratios at different scanning rates of α-MoO_3_/MXene.

To further investigate the Li^+^ storage kinetic behaviors of the α-MoO_3_/MXene composite, CV curves at various scanning rates were employed. The current contribution can be calculated from the surface-controlled behavior and diffusion-controlled processes based on CV curves, as shown in Figure 4a [22]. Referring to previous studies [15,17,20], we confirmed that the charge storage of the α-MoO_3_/MXene composites was jointly dominated by both surface-controlled pseudocapacitive behavior and diffusion-controlled battery-type behavior. The surface-controlled and diffusion-controlled contributions can be further numerically evaluated through Equation (1) [22]:

i = k_1_ν + k_2_ν^1/2^ (1)

where k_1_ν and k_2_ν^1/2^ correspond to the surface-controlled pseudocapacitive behavior and diffusion-controlled battery-type behavior, respectively. Through the above analysis, we found that the pseudocapacitive contribution (green area) accounted for 76% of the total capacity for the α-MoO_3_/MXene composite at 0.7 mV s^−1^, as shown in Figure 4b. The pseudocapacitive contribution ratio of the α-MoO_3_/MXene composite at different scanning rates is shown in Figure 4c. The pseudocapacitive contribution increased from 53% at 0.05 mV s^−1^ to 86% at 1 mV s^−1^, indicating that the diffusion-controlled contribution greatly decreased with the increase in the scanning rates and that the capacitive-controlled contribution dominated at higher scanning rates. These results show that the α-MoO_3_/MXene free-standing electrode had rapid lithiation/delithiation reaction kinetics. This can explain the excellent rate capability of the α-MoO_3_/MXene free-standing electrode.

**Figure 5:**
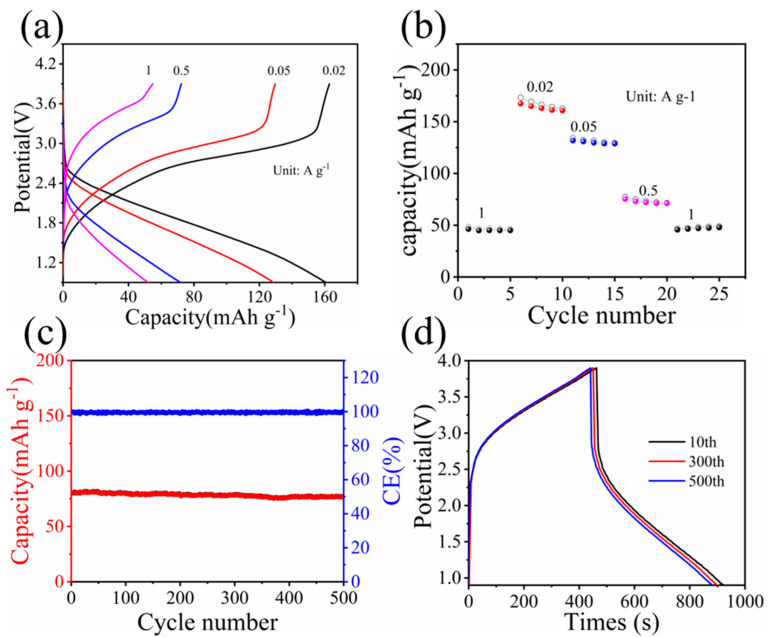
(**a**) GCD curves and (**b**) rate capability of the LiFePO_4_//α-MoO_3_/MXene full cell at different current densities. (**c**) Cycling performance of the LiFePO^4^//α-MoO3/MXene full cell in different cycles at 0.5 A g^−1^. (**d**) GCD curves of the LiFePO_4_//α-MoO_3_/MXene full cell at 0.5 A g^−1^.

Lastly, in order to evaluate the potential application of the α-MoO_3_/MXene free-standing electrode, a full cell was fabricated using the α-MoO_3_/MXene free-standing electrode as the anode and commercial LiFePO_4_ (LFP) as the cathode. The full cell exhibited a high discharge capacity of 160 mAh g^−1^ (based on the total weight of both the anode and the cathode) at a current density of 0.02 A g^−1^, and it was still able to maintain a value of 48 mAh g^−1^ at a current density of 1 A g^−1^ in a working voltage range of 0.9-3.9 V (Figure 5a,b). More importantly, the capacity of the full cell was up to 75 mAh g^−1^ with a CE of 99.8% at a current density of 0.5 A g^−1^, showing excellent cyclic stability with a high capacity retention of 97.8% after 500 continuous charge/discharge cycles (Figure 5c,d). Remarkably, an ultrahigh energy density of 284.3 Wh kg^−1^ could be achieved for our fabricated LFP//α-MoO_3_/MXene full cell at a power density of 43.3 W kg^−1^. These superior electrochemical performances of our fabricated LFP//α-MoO_3_/MXene full cell render it a highly promising candidate with both high energy and high power densities for energy storage devices in future practical applications.

## 4. Conclusions

In summary, we employed a green, cost-effective, and highly efficient strategy to rationally design an α-MoO_3_/MXene free-standing electrode to be used for the first time as the anode of a high-performance lithium-ion battery. The composites showed an obvious synergetic effect on both materials, leading to a high Li^+^ storage capacity of 1008 mAh g^−1^ at 0.1 A g^−1^, an outstanding rate capability of 172 mAh g^−1^ at 10 A g^−1^, and an excellent cycle stability for 500 cycles at 0.5 A g^−1^. This result is encouraging, considering that our α-MoO_3_/MXene electrode is entirely binder-free and carbon-free, thus making it highly similar or even superior to the previously reported Mo-based electrodes for Li-ion batteries (Table S1). Furthermore, the α-MoO_3_/MXene//LFP full cell displayed a high discharge specific capacity of 160 mAh g^−1^ at 0.02 A g^−1^ and a remarkable cycling stability (97.8% over 500 cycles at 0.5 A g^−1^). At the same time, this full cell was able to deliver a high energy density of 284.3 Wh kg^−1^ at 43.3 W kg^−1^. This excellent electrochemical performance can be ascribed to the following causes: (1) MXene acts as a conductive network and mechanical support to facilitate electron and ion transport and ensure that α-MoO_3_ provides a higher capacity, an excellent rate capability, and remarkable cycle stability; (2) α-MoO_3_ acts as a spacer to effectively prevent re-stacking between MXene-adjacent nanosheets, thereby contributing superior electrochemical properties; and (3) the free-standing electrode can obviate the need for a conductive agent, an adhesive, or heavy collectors, increasing the overall battery energy density. Owing to the excellent synergy between the two building blocks, the α-MoO_3_/MXene exhibited superior Li-ion storage performance, and it may be ranked as a promising candidate for next-generation anode materials. This strategy of fabricating an MXene-based free-standing electrode from pseudocapacitive materials is green, simple, and efficient, making it very attractive for the future construction of high-performance Li-ion batteries.

## Data Availability

The data presented in this study are available upon request from the corresponding author.

## Supplementary Materials

The following supporting information can be downloaded at https://www.mdpi.com/article/10.3390/nano12091422/s1. Figure S1: Schematic illustration of the preparation process of α-MoO_3_/MXene. Figure S2: XRD diffraction patterns of Ti_3_AlC_2_ and Ti_3_C_2._ Figure S3: (a) TEM image of Ti3C2; the inset shows the SAED pattern. (b) HR-TEM image of α-MoO_3_/MXene; the inset shows the SAED pattern. (c) HAADF image of α-MoO_3_/MXene. (d) EDS element images of α-MoO_3_/MXene. Figure S4: (a) Cycling performance of α-MoO_3_ at 0.5 A g^−1^. (b) GCD curves of α-MoO_3_ in different cycles at 0.5 A g^−^^1^. Figure S5: Rate capability at different current densities [20,36,37,38,39,40,41]. Table S1: Comparison of Li^+^ storage performances of our α-MoO_3_/MXene free-standing electrode with other reported Mo-based materials [20,36,37,38,39,40,41].
