# Supplementary material for: A Free-Standing α-MoO3/MXene Composite Anode for High-Performance Lithium Storage
Source: Nanomaterials (Basel). 2022 Apr 21;12(9):1422. doi: 10.3390/nano12091422 (PMC9104589; doi:10.3390/nano12091422)
Supplement: Supplementary file 1 [file nanomaterials-12-01422-s001.zip › nanomaterials-1617742-supplementary.pdf]

nanomaterials

Supplementary Materials

A Free−Standing α−MoO_3/MXene Composite Anode for High−Performance Lithium Storage

Zihan Guo, Dong Wang, Zhiwei Wang, Yanfang Gao*, Jinrong Liu*

School of Chemical Engineering, Inner Mongolia University of Technology, Hohhot, 010051, P.R. China; gzh@imut.edu.cn; wangd0419@163.com; zhiwei2009@126.com
*Correspondence: yf_gao@imut.edu.cn; liujr@imut.edu.cn

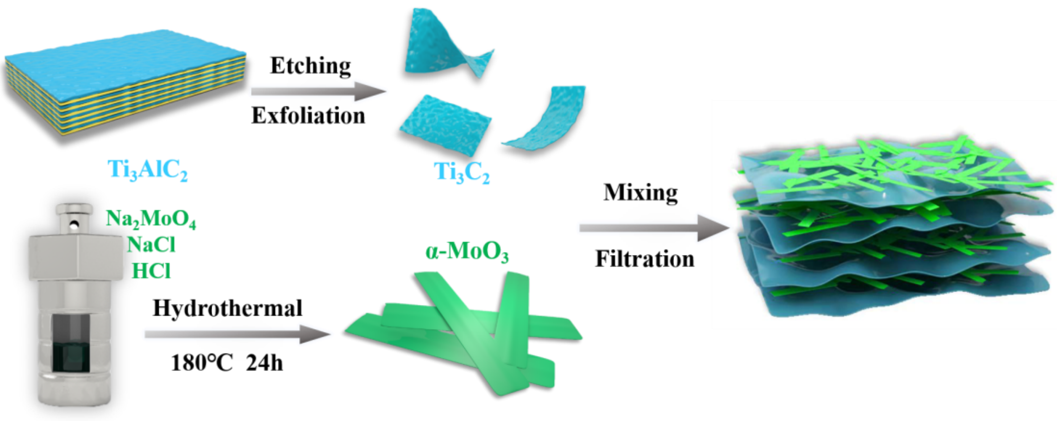

Figure S1. Schematic illustration of the preparation process of α−MoO_3/MXene.

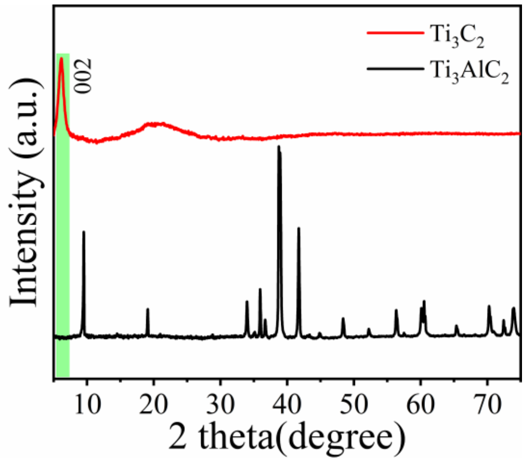

Figure S2. XRD diffraction patterns of Ti_3AlC_2 and Ti_3C_2.

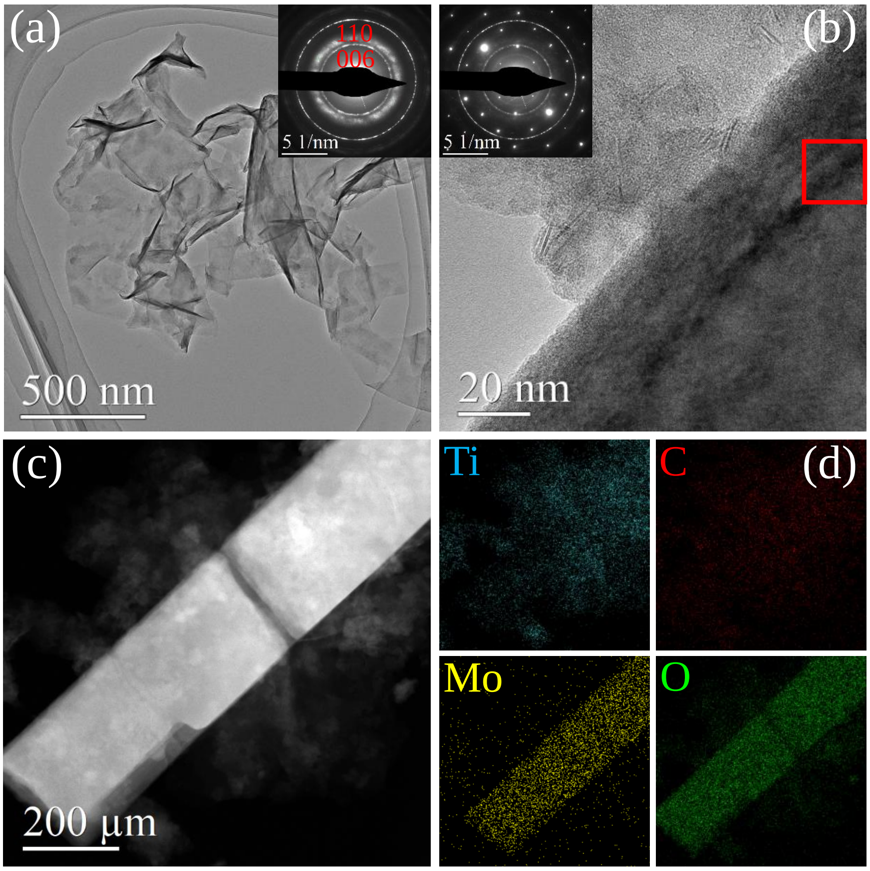

Figure S3. (a) TEM image of Ti_3C_2; the inset shows the SAED pattern. (b) HR−TEM image of α−MoO_3/MXene; the inset shows the SAED pattern. (c) HAADF image of α−MoO_3/MXene. (d) EDS element images of α−MoO_3/MXene.

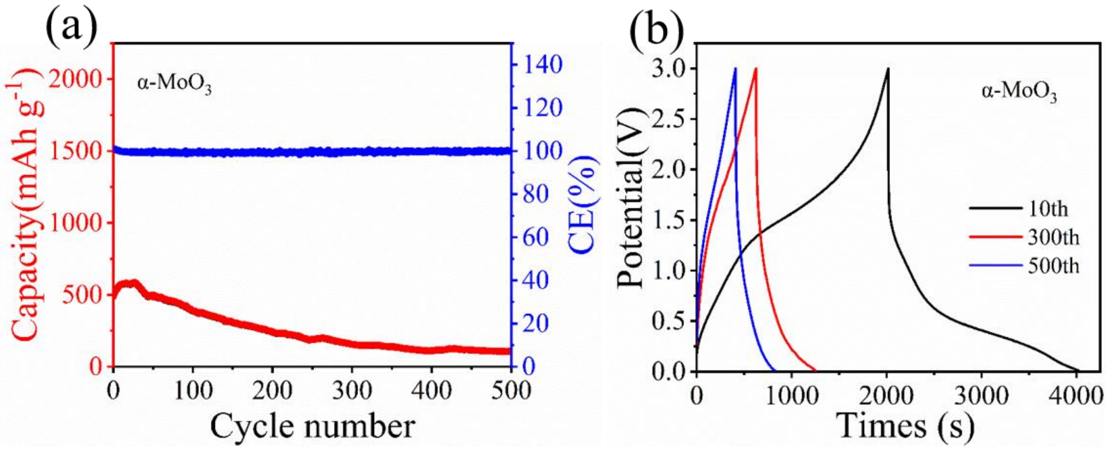

Figure S4. (a) Cycling performance of α−MoO_3 at 0.5 A g^{-1}. (b) GCD curves of α−MoO_3 in different cycles at 0.5 A g^{-1}.

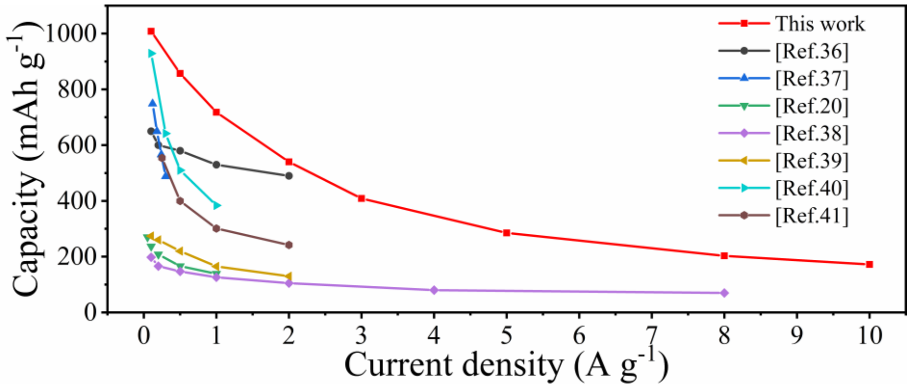

Figure S5. Rate capability at different current densities.

Table S1. Comparison of Li^+ storage performances of our α−MoO_3/MXene free−standing electrode with other reported Mo−based materials.

Materials	Current Density (A g^{-1})	Discharge Capacity (mAh g^{-1})	Rate Capability (mAh g^{-1})	Cycling Stability	Ref.
α−MoO_3@FeO_x	0.1	650	490 (2 A g^{-1})	1 A g^{-1}, 100 cycles, 110%	[36]
MoO_3@SnS_2	0.12	749	488 (0.3 A g^{-1})	1 A g^{-1},100 cycles, 53.6%	[37]
α−MoO_{3-x}@MXene	0.05	270	139 (1 A g^{-1})	0.2 A g^{-1}, 500 cycles, 78.5%	[20]
MoO_2	0.1	198	70 (8 A g^{-1})	8 A g^{-1}, 1500 cycles, 123%	[38]
α−MoO_{3-x}	0.1	274	88 (5A g^{-1})	1 A g^{-1}, 1000 cycles, 30%	[39]
MoO_3@MoS_2	0.1	929	384 (5A g^{-1})	0.1 A g^{-1}, 100 cycles, 84.1%	[40]
TiO_2−MoO_3	0.25	554	242 (2 A g^{-1})	0.25 A g^{-1}, 100 cycles, 71.0%	[41]
α−MoO_3/MXene	0.1	1008	172 (10 A g^{-1})	0.5 A g^{-1}, 500 cycles, 112%	This work
